# Occupation’s Role in Inclusion of Spanish-Speaking Older Adults in a Senior Center

**DOI:** 10.1177/15394492221093311

**Published:** 2022-05-02

**Authors:** Ryan Lavalley

**Affiliations:** 1 The University of North Carolina at Chapel Hill, USA

**Keywords:** aging, community participation, ethnography, occupation, public spaces

## Abstract

Occupation-based literature has not explored the specific community-level occupational processes that support inclusion or exclusion of Latine older adults in senior centers. This study examined occupation at the community level and its role in the inclusion or exclusion of older adult Spanish speakers in a senior center community. In addition, it sought to examine potential roles for occupational therapy practice in this realm. A 6-month ethnographic study used interviews, observations, document review, group mapping activities, and collaborative analysis to explore occupation of a senior center as the community and staff welcomed older adult Spanish speakers. Being culturally proactive, considering values and interests, offering regular activities, and cross-group doing together encouraged cohesion and communal habits of inclusion. These tailored modes of community occupation benefit Spanish-speaking older adults. Occupational therapy practitioners have opportunities to utilize tailored community occupations to support community inclusion and cohesion for this population.

## Introduction

As unprecedented human movement increases, immigrants bring with them multi-cultural enrichment and host communities have opportunities to develop systems to better serve a diversifying populace. In the United States, health inequalities for Latine elder immigrants are complex and multifaceted (Brown, 2018; Philbin et al., 2018; Tarraf et al., 2020), often perpetuated through racism in processes of everyday life (Lavalley & Johnson, 2020) and service access (Cobb et al., 2020). This population has the highest proportion of situations of poverty among immigrant groups (Lariscy et al., 2015), often experience “higher levels of functional limitations” than U.S.-born White people (Brown, 2018, p. 1527), much poorer life expectancy (Population Reference Bureau, 2013), and face “consistent and adverse disparities . . . compared with non-Hispanic Whites” (Wiener et al., 2014, p. 1).

Occupational therapy practitioners have tangible opportunities to develop systems that welcome immigrants and combat structural injustice. Occupational scientists have examined decision-making, engagement, and participation of elder immigrants through individual or agentic lenses (Johansson et al., 2013; Krishnagiri et al., 2013; Mondaca & Josephsson, 2013; Wijekoon et al., 2021; Wright-St Clair & Nayar, 2017). They have attended less to communal situations and social relationships through which these experiences are developed. None explored the specific experience of Spanish-speaking elders in the United States. As occupational researchers explore social processes of change and transformation through occupation (Bruggen et al., 2020; Lopes & Malfitano, 2020), further understandings of how communities change and how these changes affect immigrant experiences are needed.

The purpose of this article is to highlight modes of everyday community living, or occupation, that supported inclusion in a senior center welcoming Spanish-speaking elders.^1^ The study sought to examine—not cause or create—community-level occupation that was already occurring and catalyzing community change among the senior center, not just focusing on Spanish-speaker activities or perspectives.

## Theoretical Framework

I employed a transactional perspective of occupation (Cutchin & Dickie, 2013) at the communal level (Kantartzis & Molineux, 2017; Lavalley et al., 2020). This framework examines of modes of “doing together” (Lavalley, 2017)—or collective unfolding of occupation among a community (Ramugondo & Kronenberg, 2015). It examines co-production of community occupation and acknowledges this collective production is immersed in multifaceted relationships of occupation, immigration experiences, structural racism, and communities (Bailliard et al., 2021; Huot & Veronis, 2018; Mayne et al., 2016). More explicitly, this study does not seek to focus its lens on individual experiences of the community, but rather at a community-level analysis of occupation, yet still informed by individual experiences. Through this lens, the role for occupational therapy in supporting positive and health-oriented community change through “doing together” is examined (Huot et al., 2021; Lavalley et al., 2020; Lopes & Malfitano, 2020).

## Method

### Study Design

I used an ethnographic approach informed by collaborative design (Lassiter, 2005) over 6 months to capture the communal processes through which everyday doing together among staff and Spanish speakers influenced occupational opportunities for Spanish-speaking elders in a North Carolina senior center. Ethnography, which often includes participant observation, document review, interviews, participatory activities, and fieldnotes as its main data collection methods (Lassiter, 2005), is well-suited to capture multi-faceted layers of community, power, environment, politics, and occupation (Bailliard et al., 2013; Fine et al., 2003). Occupational scientists have often utilized ethnography to explore community-level occupation (Bailliard, 2013; Delaisse et al., 2021; Huot, 2013; Kantartzis & Molineux, 2014).

### Procedures

Methods within this study were approved by the University of North Carolina at Chapel Hill institutional review board and accepted by the Department on Aging administration. The study was conducted at a public senior center in North Carolina operated by a county government department on aging. The senior center is a public space open to anyone. It is in a small city within a mostly rural county. The center sits among mixed income communities and offers a variety of events, meal programs, and classes in exercise, art, crafts, Tai Chi, computers, and languages. It has strong connection to accessible and affordable transportation options and ample other services for older adults, including community occupational therapists. It acts as a major access point for services, such as support groups, home safety assessments, tax preparation, and general health and well-being support. All services were free to any older adult residents of the county.

The center was chosen because it had seen a gradual and notable increase in Spanish-speaking participants in the previous 3 to 4 years. Before this increase, participation from monolingual Spanish speakers was virtually non-existent. The site was ideal to explore how community-level occupation was facilitating—in this case successfully—support and engagement of Spanish-speaking elders. The author had previously consulted with the Department on Aging on unrelated work and had interacted briefly with regular center participants on occasion but did not have an ongoing presence in center activities.

### Participants and Consultants

All patrons, staff, and visitors of the senior center were considered participants in the study. Spanish-speaking older adults and staff members were recruited to participate more integrally in the study as *consultants* to spotlight their experiences of the community. Six staff members and seven Spanish-speaking older adults elected to be consultants. Older adult consultants were originally from Mexico, Colombia, and Puerto Rico. Six were women and one a man. Ages ranged from 66 to 82 years old. All were first-generation immigrants; some had been in the United States for only a few years and others for decades. Staff consultants held positions in varying types of roles across the organization. All staff identified as women. Four staff consultants were White. The operations manager was Black and the nutrition coordinator was a bilingual immigrant from Mexico. All had worked at the center for at least 3 years. Details about individual situations of staff or older adults, such as citizenship or economic status were not requested and only recorded when freely offered by consultants. This was also congruent with the community-level focus of the study. Highlighting the perspectives of Spanish-speaking older adults *and* staff of the community allowed for a fuller picture of the communal experience of everyday life among the center (Lavalley & Bailliard, 2020).

### Data Collection

I conducted observations and participated in activities alongside Spanish speakers and staff members in a variety of public spaces in the center, excluding staff-only areas. These included activities, such as eating lunch in the cafeteria, participating in exercise classes, sitting behind the front desk, and taking part in special events. During this data collection, I openly and broadly offered to staff and Spanish-speaking participants the opportunity to participate as consultants. Interested consultants made themselves known to me directly by their own volition. After consultants provided informed consent for more in-depth data collection methods (i.e., interviews and group mapping), I conducted one-on-one interviews with consultants. I asked them to consider how the community’s everyday life and norms had changed and were changing as more Spanish speakers came to the center.

Finally, staff and Spanish-speaking older adults gathered—in their respective groups—to complete a mapping activity where they described the activities and everyday experiences of Spanish speakers in the center. The activity used drawing materials, brochures, flyers, and other artifacts from the center to “map” the community and describe the experience of Spanish speakers in various activities and social relationships in the space. This methodology was informed by participatory mapping strategies (Parker, 2006).

### Data Analysis

I conducted all interviews and group activities in the preferred language of the consultants (English or Spanish) and transcribed them verbatim. I wrote descriptive fieldnotes, capturing everyday occupations, social interactions, environmental features, sensory experiences, and personal reflections. I also collected artifacts (e.g., event posters, brochures, newspapers) during or immediately after participant observations. I completed reflective journals regularly throughout the study. Ethnography is an iterative and reflexive methodology; analysis is not compartmentalized or disparate from data collection (Lassiter, 2005). Therefore, during data collection, I noted ideas and emerging patterns in the data and my own journals.

In addition, after all consultants were offered the opportunity, four Spanish-speaking older adult consultants chose to participate in collaborative analysis. This included a brief workshop on coding, reading transcripts from their own interview and the group mapping activity, and a follow-up workshop to discuss and develop themes from the data. These initial themes were the foundation on which I continued further and more structured thematic analysis (Saldaña, 2013). This analysis was integrated with a broader ethnographic analysis (Lassiter, 2005). Data beyond individual interviews, such as participant observations, artifacts, and group activities, revealed the reification of policies, social norms, and routines rooted in community relationships rather than individual perspectives.

## Findings

Below, I describe the specific modes through which the community “did together” across staff and Spanish speakers that were particularly salient in supporting or deterring inclusion for Spanish-speaking elders.

### Being Culturally and Linguistically Proactive

The established expectation in the center was that older adults, no matter their preferred language, would seek out staff members to request services, modifications, or increased accessibility, of any kind, to occupations in English. Community norms tacitly held English as the default language:

Esta gente no cree que ese viene por estos hispanos que no saben inglés. “Vamos a colocar ese en español.” Nada. Vaya a mirar a la puerta dónde nosotros entramos. Todo en inglés. Y eso que dicen varias . . . puedo leer. Pero otros nada. No sabe qué dice . . . (Esmeralda, Adulta Mayor)

[These people do not believe that this is for those Hispanics who do not know English. “We are going to hang that in Spanish.” Nothing. Go look at the door where we enter. Everything in English. And several say . . . I can read. But others nothing. One does not know what it says . . . (Esmeralda, Older Adult)]

Non-Spanish-speaking staff were not accustomed to considering the many logistical barriers in accessing occupational opportunities when one only spoke Spanish—often rooted in historical racism and discrimination against immigrants (Armenta, 2017; Bailliard et al., 2021). While not purposeful or malicious, the center’s everyday life was saturated with these problematic and often unnoticed norms. The following comments from staff exemplify this:

I’m not fortunate enough to learn Spanish.

And it’s unfortunate, if you will, that more of us don’t take the time or can’t commit to [lowers voice a bit] learning multiple languages.

Interviewer: How do you think that would look? How do you think they would come to the staff [to access services]?

Staff: I don’t know because I don’t speak Spanish, but I want them, if they have a complaint, I want them to be able to learn how to voice it, or how we can help . . .

Therefore, often non-Spanish-speaking staff did not see it as their responsibility to proactively mitigate barriers to occupational opportunities.

Yet, Spanish speakers were not exempt from this norm. Older adults often assumed that English was necessary to participate fully in the center:

Porque si no sabes el idioma, ¿qué haces aquí entre un montón de gente que no te van a entender o que no te puedes comunicar? Y sí, sí es importante que estamos en un país dónde el idioma oficial es inglés. Todo el mundo hace fuerza por aprender. Yo pienso que es muy importante eso. (Marta, Adulta Mayor)

[Because if you do not know the language, what are you doing here among a lot of people who will not understand you or who cannot communicate? And yes, it is important that we are in a country where the official language is English. Everyone makes the effort to learn. I think that is very important. (Marta, Older Adult)]

Although English is not the official language of the United States, this implicit social norm assumed Spanish speakers should adapt to and navigate English-based systems rather than the center community proactively offering Spanish information.

When information was proactively offered, Spanish speakers connected to more occupations. For example, a bilingual social worker publicized an open house event in Spanish-speaking media with assurance that Spanish-speaking staff and volunteers would be available. Caregivers and older adults attended, and many began participating in the center regularly.

### Considering Relevance and Value

Staff were interested in “being sensitive or showing more that we do care” (Dorothy, Staff) by attempting to offer targeted activities for diverse groups, such as culturally celebratory events around *Día de los Muertos* festivities or *Cinco de Mayo*. While older adults who were Mexican appreciated these, many Spanish speakers did not connect, preferring celebrations relevant to their origin countries. Furthermore, activities, such as exercise and health education in Spanish were more appealing in general:

A mi me gustaría que todos viniéramos y hiciéramos ejercicio diario. Eso me encantaría. Porque pues, todos estaríamos más saludables y ya menos al medico y estaríamos más felices. (Marta, Adulto Mayor)

[I would like everyone to come and do daily exercise. I would love that. Because well, we would all be healthier and go to the doctor less, and we would be happier. (Marta, Older Adult)]

When the center hosted a weekly “Spanish social club” where Spanish speakers participated in exercise, education, and social outings led by a bilingual social worker and a Spanish-speaking elder volunteer, elders from across county lines came to participate. Cristina compared this senior center to one closer to her, “Está el grupo para que vino. Me gusta más acá. Allá, era de inglés nada más. [Here is the group to come to. I like here better. There, it was only in English.]” Prior to this group, few activities or services in Spanish were offered in general, let alone focused on their specific interests and values. When the community proactively facilitated occupations in Spanish that connected with everyday interests of Spanish-speaking older adults, more participants than ever attended.

### Being Regular and Consistent

Older adult consultants appreciated the regularity of the social group and other scheduled occupational opportunities because they could rely on a comfortable, accessible space with others who spoke their language and shared similar cultural backgrounds. Multiple older adult consultants described how this regularity countered uneasiness when entering the center for the first time:

En el principio, como no veíamos gente, entonces no veníamos . . . (Rubí, Adulta Mayor)

[In the beginning, since we weren’t seeing people, we weren’t coming . . . (Rubí, Older Adult)]

Siempre uno viene con miedo porque nadie hablaba español . . . cuando yo llegue, pues estaba [la trabajadora social]. Muy linda ella con nosotros. Eso nos dio como seguridad, porque ella hablaba español. (Esmeralda, Adulta Mayor)

[Always one comes with fear because no one spoke Spanish . . .When I arrived, well there was [the social worker]. Very nice, to have her with us. That gave us like security, because she spoke Spanish. (Esmeralda, Older Adult)]

Primero es un choque cultural. Entras y pues ves allí puros güeros, pura gente americana o African Americans. Pues no ves gente de tu raza. Es para todos lados, estoy solita en este lugar ¿no? Sí, es un choque. (Marta, Adulta Mayor)

[First, it is a cultural shock. You enter and well, you see purely blond people, purely American people, or African Americans. Well, you don’t see people of your race. It’s everywhere. I am alone in this place, no? Yes, it’s a shock. (Marta, Older Adult)]

Spanish speakers could reliably expect peers, staff, and occupations that were accessible in Spanish without having to search for varying times or locations in mostly English information. Attending the social group became routine much more easily for older adults and caregivers.

Consistency fostered community and relationship building among Spanish speakers. In spaces held for and by Spanish speakers, social norms, greetings, and habits shifted to match their preferred ways of doing together. Francisco shared that the social club offered a place where he could “sentirse en el ambiente del idioma [to feel in the environment of the language]”. Facing significant anti-immigrant and anti-Latine racism in the broader culture, this offered a welcoming landing pad for further participation in the center community.

These consistent and reliable opportunities fostered a strong foundation through which Spanish speakers and staff could venture further in the community. Francisco also reminisced on learning how the front desk functioned:

En el transcurrir del tiempo, hemos aprendido. . .como funciona. Entonces, en el “front desk” hay un pizarrón o un tablero en dónde uno tiene que pasar a leer las actividades, ¿verdad? En qué horario y qué salón hay para uno pueda asistir, integrarse, participar.

[In the passing of time, we have learned . . . how it functions. So, at the “front desk” there is a whiteboard or board where one has to pass by and read the activities, right? What time and what room, so one can attend, integrate, and participate.]

The community at large became more familiar with Spanish speakers’ presence, and they began to explore more activities not in Spanish. Particularly exercise, jewelry making, and Tai Chi classes were popular because of visual guidance in activities.

### Doing Together Across the Community

Leaders of the group introduced some staff, such as the transportation specialist, community occupational therapist, and social worker as part of educational workshops, often translated by the bilingual social worker. Spanish speakers also began attending some center-wide special events where staff were present. Doing together with staff fostered familiarity, or a “felt presence,” as Lacy, one staff consultant put it. This felt presence often spurred staff to be more responsive.

Lisa, the transportation specialist emphasized the importance of “hanging out” with Spanish speakers to more effectively offer opportunity to connect with services offered. She, a non-Spanish speaker, was well known to Spanish speakers because, since giving an educational program, she often purposefully paused in the hall just to connect with the group. Staff and Spanish speakers “did together” and gained familiarity across the community.

### Ongoing Barriers

At first, both staff and Spanish speakers relied heavily on the bilingual social worker and the occupations of Spanish speakers were somewhat isolated. The social group did not typically explicitly facilitate participation in activities beyond their scheduled routine. Thus, connections to staff or other services were less frequent when the bilingual social worker left the position and it was not refilled. Spanish speakers often did not feel comfortable asserting themselves into other spaces or activities, preferring to wait to be invited. Some expressed not wanting to be a “burden” while others did not yet feel the center was “their community.” This limitation was less severe but still present as Spanish speakers became more familiar with the center community.

### Discussion

The findings of this study revealed that communities that are being culturally and linguistically proactive, considering the relevance and value of occupations for specific populations, offering those activities regularly and consistently, and doing together in everyday practice can potentially contribute to more inclusive communities for Spanish-speaking elders. Occupational scientists have highlighted, in other settings, the role of occupation in creating social norms, spaces, and cultural movements, particularly from the perspective of groups within a community (Kantartzis & Molineux, 2017; Simaan, 2017). Studies of other linguistic minorities have emphasized the nuanced ways in which immigrants manage and develop new spaces, mostly among themselves (Delaisse et al., 2021; Johansson et al., 2013; Wijekoon et al., 2021). Yet, few have highlighted the role of non-immigrants in community occupation in response to immigration.

However, the findings of this study may not capture the extent to which structural racism (Lavalley & Johnson, 2020) and culturally exclusive practices outside the center were also contributing to the experiences of staff and older adults. Spanish speakers’ experience cannot be separated from structural injustices in broader policies or social contexts (Bailliard, 2013). In addition, the somewhat acquiescent demeanor of some older adults in this study is not necessarily congruent with the renewal and generativity of later life seen elsewhere in the literature (Lawson, 2000; Wijekoon et al., 2021).

Yet, this study highlights potential value of facilitating everyday activities that encourage non-Spanish and Spanish speakers to engage together in positive ways. It offers an instance where these interactions facilitated positive community outcomes, however, also further exemplifies moments when everyday life can be a conduit for reinforcing discriminatory structures and norms (Lavalley & Johnson, 2020). Therefore, vigilance and tailored examination of everyday living for different communities who are welcoming different immigrant groups is always necessary. In the specific socio-political context of this study, these collective strategies were effective in supporting inclusion for Spanish-speaking older adults.

### Limitations

This study was limited by time and capacity to engage with more populations within the senior center beyond Spanish speakers and staff members. In addition, I am not a native Spanish speaker, therefore, some nuance and depth of cultural knowledge may have been lost in data related to Spanish-speaking adults, however, experience as a community occupational therapist positioned me well to have a deeper understanding of staff perspectives. A further limitation was the lack of staff involvement in collaborative analysis.

## Implications for Occupational Therapy Practice

This study firmly situates community-level processes in relation to occupation, further connecting the re-emerging role of occupational therapy practitioners in facilitating community-level—rather than only individual-level—processes of everyday life (Fritz & Cutchin, 2017). This study contributes to developing models of community-level occupational therapy practice that are specifically rooted in collective, communal, or social perspectives (e.g., Galvaan & Peters, 2017; Laliberte-Rudman et al., 2018; Lopes & Malfitano, 2020). These findings conceptualize *how* occupational therapy practitioners could leverage occupation through community programs, social movements, and policy change to influence community-level outcomes—rather than aggregated individual measures (e.g., Estrany-Munar et al., 2021). This evidence offers actionable mechanisms for occupational therapy practitioners in community organizing and social transformation with this population. In this case, policies, environment, and everyday living constructed obstacles based on intersectional systems of power, racism, and language norms. However, the modes of doing together described in this article disrupted racist norms and barriers, facilitating positive relationship building and increased accessibility among the community, as well as specific policy changes (Lavalley et al., 2020). Therefore, facilitating occupational experiences at the community level, such as these is one tool occupational therapy practitioners have in contributing to more equitable and just communities.

## Conclusion

These lessons highlight the potential for situations of everyday life to be mobilized for community change in ways that support inclusion, health, and justice, emphasizing the potential role for occupational therapy practitioners in these realms. Community occupation is an important tool in fostering inclusive communities and an occupational lens is useful in developing processes and programs that can support these efforts. While these lessons may not be applicable to all communities welcoming linguistic minorities or even all senior centers and older adult programs, further research could explore the most beneficial ways to mobilize these aspects of occupation in other community spaces. Findings from this study offer valuable considerations for occupational therapy practitioners who are working to support inclusive communities, particularly for a diverse older adult population.

## Notes

1. During this study, Spanish-speaking consultants arrived at no clear consensus on how or if they preferred to be labeled as a group. Often rejecting monolithic labels (e.g., Hispanic, Latinx), and preferring to identify with origin countries (e.g., Mexican, Colombian). However, they still differentiated themselves from other groups, recognizing some shared cultural norms. Therefore, I refer to older adult consultants as “Spanish-speaking” because it was truly the social element that gathered them. Further, this label does not rely on a racialized categorization.
